# *Chlamydia pneumoniae* Influence on Cytokine Production in Steroid-Resistant and Steroid-Sensitive Asthmatics

**DOI:** 10.3390/pathogens9020112

**Published:** 2020-02-11

**Authors:** Dóra Paróczai, Tímea Mosolygó, Dávid Kókai, Valéria Endrész, Dezső P. Virok, Attila Somfay, Katalin Burián

**Affiliations:** 1 Department of Medical Microbiology and Immunobiology, University of Szeged, Dóm sqr. 10., 6720 Szeged, Hungary; paroczai.dora@med.u-szeged.hu (D.P.); mosolygo.timea@med.u-szeged.hu (T.M.); kokai.david@med.u-szeged.hu (D.K.); endresz.valeria@med.u-szeged.hu (V.E.); virok.dezso.peter@med.u-szeged.hu (D.P.V.); 2 Department of Pulmonology, University of Szeged, Alkotmány str.36., 6772 Deszk, Hungary; somfay.attila@med.u-szeged.hu

**Keywords:** *C. pneumonia*, cytokine, asthma, steroid-resistant, infection

## Abstract

Medications for asthma management consisting of inhaled corticosteroids act by controlling symptoms. However, some patients do not respond to steroid treatment due to immunological factors at the cytokine level. *Chlamydia pneumoniae* (*C. pneumoniae*) infection is strongly implicated in asthma pathogenesis, causing altered immune responses. We investigated the association of *C. pneumoniae* serostatus with the production of certain cytokines by peripheral blood mononuclear cells (PBMCs) of steroid-resistant and -sensitive asthmatic patients. Our most important findings are the following: In the case of *C. pneumoniae* seropositive patients we detected pronounced spontaneous interleukin (IL)-10 secretion and, in the case of steroid-resistant patients, IL-10 secretion was at a significantly higher level as compared with in-sensitive patients (*p *< 0.01). Furthermore, steroid-resistant seropositive patients produced a significantly higher level of IL-10 spontaneously and under antigen stimulation as compared with steroid-resistant seronegative individuals (*p *< 0.05). Concerning spontaneous TNF-α secretion by *C. pneumoniae* seropositive asthmatics, we observed that steroid-resistant patients produced significantly more of this cytokine than steroid-sensitive patients. In the steroid-resistant patients’ sera, a remarkably high MMP-9 concentration was associated with *C. pneumoniae* seronegativity. Our study revealed that the differences in the cytokine production in steroid-sensitive and -resistant asthmatic patients can be influenced by their *C. pneumoniae* serostatus.

## 1. Introduction

Asthma is a chronic airway disease associated with airway remodeling, reversible bronchial obstruction, and airway hyperresponsiveness (AHR), and 1% to 18% of the global population are affected. Approximately 5% to 10% of asthmatic patients fail to fully respond to steroid therapy, and these patients also have higher mortality and morbidity rates [1]. A diagnosis of steroid resistance is made when patients exhibit <15% improvement in the forced expiratory volume in one second (FEV1) during post-bronchodilator spirometry after 14 days of oral prednisolone therapy. Steroid resistance was first recognized in 1968 and several studies have since been performed in order to expand our understanding of the complex mechanisms resulting in steroid insensitivity [2]. Currently, there is an emerging clinical need to identify the factors contributing to the disease pathogenesis, as severely steroid-resistant asthmatic patients do not respond well to conventional therapies. A variety of factors such as infections and air pollution cause changes in cytokine production at the transcriptional and protein level, leading to steroid resistance [3,4,5]. Several studies have confirmed that infections, particularly early-life respiratory infections, are implicated in the pathogenesis of steroid-resistant asthma [6,7,8].

*Chlamydia pneumoniae* (*C. pneumoniae*) causes community-acquired pneumonia and accounts for airway remodeling in chronic lung diseases [9]. Asthmatics with earlier *C. pneumoniae* infection are more likely to develop steroid-resistant asthma, and their positive serostatus is associated with an increased severity of asthma and airway neutrophilia [10,11]. 

Interleukin (IL)-10 is an anti-inflammatory cytokine produced by regulatory T cells, macrophages, and even dendritic cells. It plays a crucial role in maintaining lung immune responses and participates in asthma pathogenesis by regulating and inhibiting Th2 responses. Asthmatic patients exhibit diminished IL-10 production in bronchoalveolar lavage (BAL) fluids and, to the best of our knowledge, there are no congruent data about the IL-10 production by the peripheral blood cells of these patients. In addition, IL-10 secretion by circulating cells has not been investigated in relation to the use of inhaled corticosteroids [12,13]. We hypothesized that a previous *C. pneumoniae *infection can change cytokine pattern and have an impact on subsequent IL-10 production with or without specific antigen stimulation of cells from steroid resistant asthmatics. IL-10 regulates responses of immune and airway cells that are infected by *C. pneumoniae*. A former, persistent infection can affect cytokine production through enhancing Toll-like receptor (TLR) signaling and nucleotide-binding oligomerization domain-like receptor family, pyrin domain-containing 3 (NLRP3) activity. TLR2, 4 signaling, and NLRP3 activity have a connection with glucocorticoid resistance mechanisms, including changes in glucocorticoid receptor (GR) expressions and altered cytokine secretion [14,15]. To the best of our knowledge, there are no data about the differences in cytokine production between asthmatics related to *Chlamydia* serostatus. Consequently, we hypothesized that determining IL-10 responses in steroid-resistant and -sensitive asthmatics proves that a former infection induces alterations in a different manner in asthma phenotypes. 

Tumor necrosis factor alpha (TNF-α) responses play a significant role in AHR via eosinophil and neutrophil attraction, nuclear factor kappa B (NF-κB) activation, production of adhesion molecules, and even myocyte proliferation [16]. All of these factors, along with immune and cytokine responses, can lead to the modification of GRs and changes in receptor affinity and binding capacity, resulting in reduced steroid responsiveness and a decline in lung function [17]. *C. pneumoniae* is able to induce TNF-α production and trigger cellular proliferation, leading to decreased steroid responsiveness of peripheral blood mononuclear cells (PBMCs) [18]. A previous *C. pneumoniae* infection could have a long-term effect on TNF-α response, hence, we investigated TNF-α secretion by PBMCs of *Chlamydia*-specific IgG negative and positive patients.

Matrix metalloproteinases (MMPs) and their inhibitors are involved in the changes of the extracellular matrix and determine airway epithelium thickness. MMP-9 has a pivotal role in remodeling and was the first to be investigated in asthma Elevated MMP levels, particularly MMP-9, are detected in the BAL fluids and even in the sera of asthmatic patients [19,20]. Rödel et al. found increased MMP-1 and -3 production due to *C. pneumoniae* infection in smooth muscle cells [21]. *C. pneumoniae* affects MMP-9 and tissue inhibitor of metalloproteinase-1 (TIMP-1) production by PBMCs and weakens the impact of glucocorticoids on the secretion of MMPs [22]. The levels of MMP-9 inhibitor, TIMP-1, can be altered in asthmatics, however, the relationship with infections are not well studied. As corticosteroids do not normalize the elevated MMP-9 levels [23], we hypothesized that *C. pneumoniae* infection has a long-term effect in asthmatics and can lead to differences in MMP-9 level between steroid-resistant and -sensitive patients. There are no data regarding this association. MMP-9 seemed to be differentially released in exhaled condensates from asthmatics and based on this phenomenon, we can determine different biological phenotypes of asthma that can help to monitor diseases severity [24]. On the basis of the above-mentioned results, our aim was to compare MMP-9 levels in steroid-resistant and -sensitive asthmatic patients’ sera which could contribute to a better understanding of steroid-resistant asthma features.

Taken together, IL-10 and TNF-α cytokine production by PBMCs of steroid-sensitive and -resistant asthmatic patients have not been analyzed without and with antigen stimulation in relation to their *C. pneumoniae* serostatus. As *C. pneumoniae* is involved in asthma exacerbation, as well as in persistent infections, it can have a momentous impact on the cytokine production in asthmatic patients. The long-term effects of chronic *C. pneumoniae* infection on cytokine production in patients with asthma remain unclear. MMP-9 is implicated in the remodeling process of the lung and is believed to be influenced by *C. pneumoniae* infection. As there are no data available regarding MMP-9 levels in steroid-sensitive and -resistant asthmatics, we intended to define differences in the patients’ sera according to the *C. pneumoniae* serostatus and steroid responsiveness. The primary aim of this research was to find differences in steroid-resistant and -sensitive patients related to *C. pneumoniae* serostatus. 

## 2. Results

### 2.1. Patient Characteristics and Demographics

In this study, 40 steroid-sensitive asthmatic patients (65% female, 35% male, with a mean age of 59 years) and 40 steroid-resistant asthmatic patients (68% female, 32% male, with a mean age of 63 years) were enrolled. Steroid resistance was defined by the following criterion: Patients did not achieve >15% improvement in the FEV1 value after 14 days of oral prednisolone (40 mg/day) therapy. In accordance with our expectations, the steroid-resistant group exhibited significant differences in dynamic lung volumes (Table 1). The steroid-resistant group had a mean FEV1 value of 56% ± 0.2%, with a significant difference as compared with the sensitive group with a mean FEV1 value of 72% ± 0.22% (*p = *0.01). The ICSs used were budesonide/formoterol dry powder inhaler (daily doses ranging from 400 to 1280 µg), cyclesonide hydrofluoroalkane (HFA) (daily doses ranging from 320 to 640 µg), fluticasone propionate/salmeterol HFA (daily doses ranging from 500 to 1000 µg), and beclomethasone dipropionate/formoterol HFA (daily doses ranging from 400 to 1000 µg). In the sensitive group, 50% of patients received high daily ICS doses, while in the resistant group, 95% of patients used high dose ICS. Steroid doses were determined according to the GINA guideline [1]. Further clinical characteristics of asthmatic patients are provided in Table 1. As controls, 40 non-asthmatic, healthy blood donors were selected. 

BMI, body mass index; CHD, coronary heart disease; FEF 25/75, forced expiratory flow at 25% to 75% of the pulmonary volume; FEV1, forced expiratory volume in 1 s; FVC, forced vital capacity; ICS; inhaled corticosteroid; LABA, long-acting β-agonist; LTI, leukotriene inhibitor; py, pack year; SABA; short-acting β-agonist.

### 2.2. C. pneumoniae-Specific Serological Status of Asthmatic Patients

**Figure 1:**
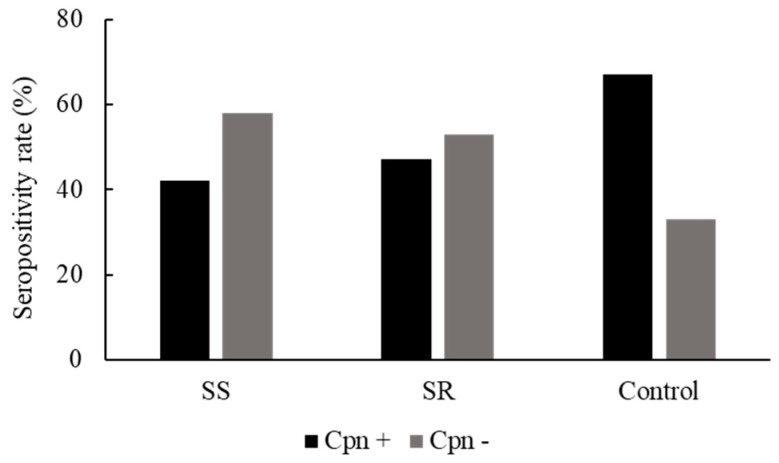
*C. pneumoniae* serostatus of healthy blood donors (controls) and patients with asthma. *C. pneumoniae* serostatus was determined by an enzyme-linked immunosorbent assay from the native blood samples from controls and steroid-sensitive (SS) and steroid-resistant (SR) asthmatics (*C. pneumoniae* seropositive (Cpn+) and *C. pneumoniae* seronegative (Cpn−)).

First, we assayed the serum samples for the presence of *C. pneumoniae*-specific IgG to determine the seropositivity rate in each group. The control group representing the average Hungarian population exhibited a 67% seropositivity rate. Surprisingly, we observed a lower *C. pneumoniae* seropositivity rate in asthmatic patients than among the controls. In asthmatic patients, 42% of steroid-sensitive and 47% of steroid-resistant participants were *C. pneumoniae *IgG-positive (Figure 1).

### 2.3. IL-10 Cytokine Production in Asthmatics in Response to Specific (C. pneumoniae) and Nonspecific phytohemagglutinin (PHA) Stimulation

PBMCs obtained from steroid-resistant and -sensitive asthmatics were cultured with the *C. pneumoniae* antigen or phytohemagglutinin (PHA); or were untreated. 

**Figure 2:**
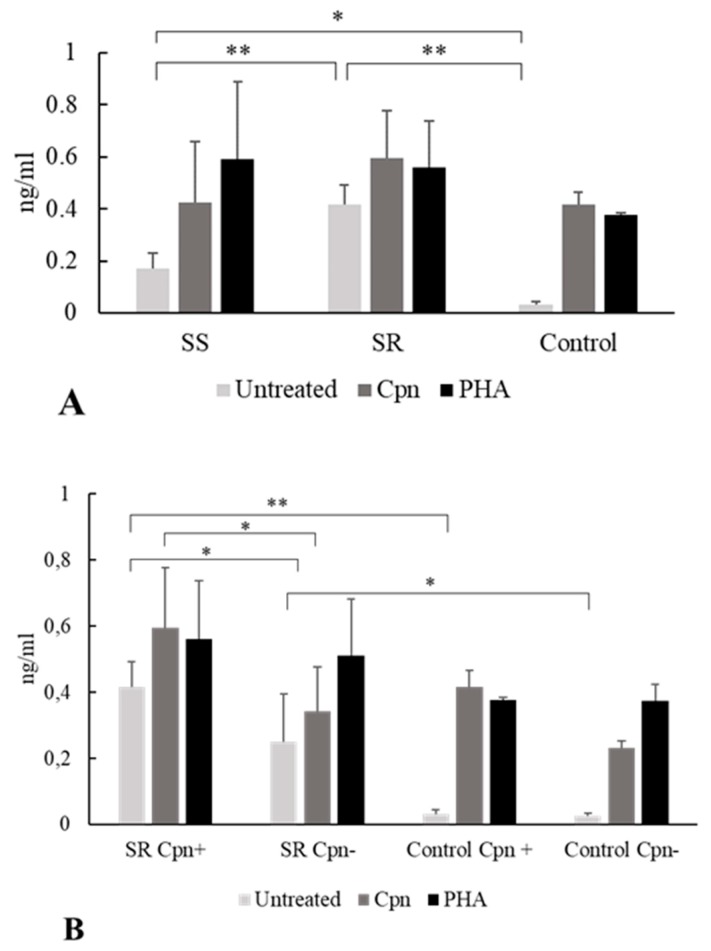
Effect of stimulation with *C. pneumoniae* and phytohemagglutinin (PHA) on interleukin (IL)-10 secretion. Human peripheral blood mononuclear cells (PBMCs) were obtained from steroid-resistant (SR) and steroid-sensitive (SS) asthma patients and healthy blood donors (controls) with *C. pneumoniae *seropositivity (Cpn+) and seronegativity (Cpn−) as follows: (**A**) IL-10 concentrations were determined by enzyme-linked immunosorbent assay and expressed as ng/mL (mean ± SD) in the *C. pneumoniae* seropositive groups. (**B**) IL-10 production by PBMCs of *C. pneumoniae* seropositive and seronegative SR patients as compared with controls (mean ± SD). Asterisks indicate significant differences (* *p *< 0.05, ** *p *< 0.01).

Untreated PBMCs from *C. pneumoniae* seropositive, steroid-sensitive patients secreted a significantly higher amount of IL-10 than did those from the *C. pneumoniae* positive non-asthmatic blood donors (0.17 ± 0.06 ng/mL versus 0.03 ± 0.01 ng/mL,* p = *0.04). The same tendency was observed in the seropositive steroid-resistant group, as their PBMCs spontaneously produced a higher amount of IL-10 than did those of seropositive non-asthmatic blood donors (0.41 ± 0.07 ng/mL versus 0.03 ± 0.01 ng/mL,* p = *0.0002) (Figure 2A). 

Moreover, significantly higher IL-10 production was detected without stimulation in *C. pneumoniae* seropositive, steroid-resistant patients than in steroid-sensitive patients (0.41 ± 0.07 ng/mL vs. 0.17 ± 0.06 ng/mL,* p = *0.002) (Figure 2A). 

Interestingly, when the PBMCs from *C. pneumoniae* seropositive individuals were cultured with *C. pneumoniae* antigen or PHA, no significant difference was observed between the asthmatic groups and the control group related to IL-10 production (Figure 2A).

Concerning steroid resistance, we compared IL-10 production in *C. pneumoniae *seropositive and seronegative asthmatics. We found that in cases of seropositivity, steroid-resistant patients exhibited significantly higher spontaneous IL-10 cytokine release than did seronegative individuals (0.41 ± 0.07 ng/mL vs. 0.25 ± 0.14 ng/mL, *p *= 0.02). Moreover, after specific *C. pneumoniae* antigen stimulation, significantly higher IL-10 levels were found in the seropositive steroid-resistant group than in the seronegative steroid-resistant group (0.59 ± 0.18 ng/mL vs. 0.34 ± 0.13 ng/mL, *p = *0.02). Regarding the cytokine response to nonspecific PHA stimulation, we observed no differences related to the *C. pneumoniae* serostatus among steroid-resistant asthmatics (Figure 2B). 

### 2.4. TNF-α Production in Asthmatics in Response to Specific (C. pneumoniae) and Nonspecific PHA Stimulation

**Figure 3:**
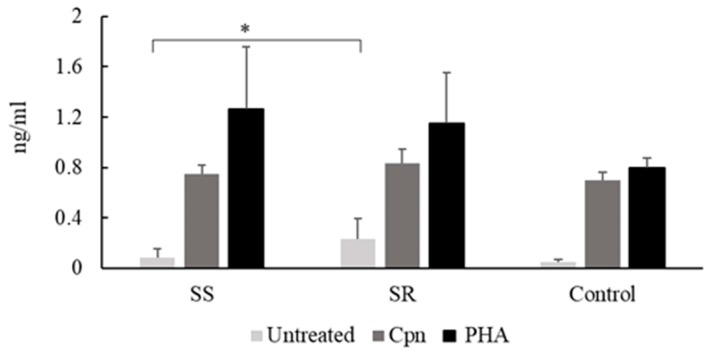
*C. pneumoniae* and PHA induced TNF-α production of PBMCs from seropositive control and asthmatic participants. * *p *< 0.05.

PBMCs obtained from asthmatics and cultured without stimulation produced a higher amount of TNF-α than did those from non-asthmatics, however these differences did not reach significance. PBMCs from *C. pneumoniae* seropositive steroid-resistant participants spontaneously secreted a higher level of TNF-α than did seropositive steroid-sensitive patients (0.23 ± 0.16 ng/mL vs. 0.08 ± 0.06 ng/mL,* p *= 0.05) (Figure 3).

In response to specific and nonspecific treatments, in *C. pneumoniae* seropositive asthmatics there was no significant difference in the secreted TNF-α levels between the steroid-sensitive and -resistant groups.

Tumor necrosis factor-alpha (TNF-α) content of the supernatants of peripheral blood mononuclear cells (PBMCs) from seropositive steroid-sensitive (SS) and steroid-resistant (SR) asthmatic patients and healthy blood donors (controls). TNF-α concentrations were determined by enzyme-linked immunosorbent assay and expressed as ng/mL (mean ± SD). Significant differences are labelled with asterisks, * *p *< 0.05.

### 2.5. MMP-9 Production in Steroid-Sensitive and Steroid-Resistant Asthmatic Patients

**Figure 4:**
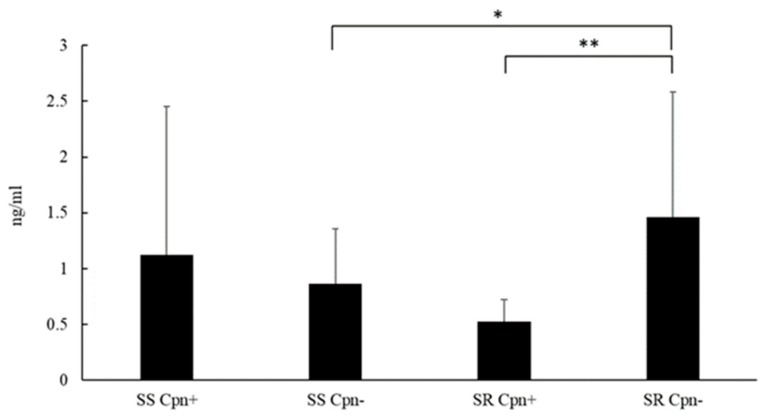
Serum matrix metalloproteinase (MMP)-9 levels in patients with asthma. * *p *< 0.05, ** *p *= 0.01.

The serum level of MMP-9 was measured in *C. pneumoniae* seropositive and seronegative steroid-sensitive and steroid-resistant asthmatics. A significant difference in the serum MMP-9 level was observed among the steroid-resistant participants. *C. pneumoniae* seronegative patients exhibited significantly increased serum levels of MMP-9 as compared with those found in *C. pneumoniae* seropositive asthmatics (*p *= 0.01, 1.46 ± 1.124 ng/mL vs. 0.528 ± 0.193 ng/mL) However, this difference was not observed among steroid-sensitive patients. In association with *C. pneumoniae* seronegativity, a statistically significantly higher MMP-9 level was found in the sera of steroid-resistant patients than in steroid-sensitive patients (1.46 ± 1.125 ng/mL vs. 0.87 ± 0.49 ng/mL,* p *= 0.04) (Figure 4). 

MMP-9 levels were measured in steroid-sensitive (SS) and steroid-resistant (SR) *C. pneumoniae* seropositive (Cpn+) and seronegative (Cpn−) asthmatic patients using an enzyme-linked immunosorbent assay. Data are expressed as ng/mL (mean ± SD). Significant differences are labelled with asterisks, * *p *< 0.05 and ** *p *= 0.01.

## 3. Discussion

Asthma is a heterogeneous, reversible, obstructive lung disease with systemic immunological features and variable phenotypes. In this study, we examined cytokine responses in asthmatic patents with different asthmatic phenotypes in relation to their steroid responsiveness and *C. pneumoniae* serological status. We aimed to determine whether steroid-resistant asthmatics and steroid-sensitive patients differ only in treatment responsiveness and clinical features, or in the cytokine response to the presence of specific antigens as well. Therefore, we investigated the in vitro IL-10 and TNF-α responses of *C. pneumoniae* seropositive and seronegative steroid-resistant and steroid-sensitive asthmatic patients’ PBMCs to a polyclonal mitogen and *C. pneumoniae* antigen. Moreover, we examined MMP-9 blood serum levels in association with their *C. pneumoniae *serostatus to detect differences between steroid-resistant and -sensitive asthmatics. 

We hypothesized that a prior *C. pneumoniae* infection has an impact on IL-10 production in asthmatic patients. We examined cytokine production under different stimuli in steroid-resistant and steroid-sensitive patients. Untreated PBMCs from *C. pneumoniae* seropositive patients secreted a higher level of IL-10 than did those from controls. These data correspond to the fact that higher serum IL-10 levels were observed in asthmatics than in controls [25]. Our results indicated that increased IL-10 responses are derived from PBMCs, indicating that in asthma pathogenesis, systemic immune responses can alter disease severity and clinical features. 

In the *C. pneumoniae* seropositive groups, we detected greater spontaneous IL-10 secretion in steroid-resistant individuals than in steroid-sensitive individuals. Additionally, PBMCs from steroid-resistant *C. pneumoniae* seropositive asthmatics produced significantly higher IL-10 responses when untreated and under *C. pneumoniae *stimulation, supporting the notion that earlier *C. pneumoniae* infection contributes to an altered IL-10 response in steroid-resistant asthmatics. In contrast, we did not observe a similar tendency in steroid-sensitive patients, reflecting the distinct immunological features in steroid-resistant participants. 

In asthmatics, there is a positive correlation between disease severity and the IL-10 level. Accordingly, patients with asthma exhibit a higher IL-10 serum level than that of healthy individuals [26]. Glucocorticoids enhance IL-10 secretion and Treg functions, consequently, in steroid-resistant asthmatics, these effects cannot be observed clearly. However, it is well-known that IL-10 responses can be reversed and steroid responsiveness can be repaired in steroid-resistant asthmatics [27]. The precise role of IL-10 is unclear in asthma pathogenesis, particularly, in steroid-resistant asthmatics, and appears to be pleiotropic. One study revealed that under dexamethasone stimulation, CD4+ T cells from patients with steroid-resistant asthma failed to induce IL-10 synthesis [28]. To the best of our knowledge, IL-10 production by PBMCs from asthmatic patients under different stimuli have not been studied in relation to the *C. pneumoniae* serostatus and steroid responsiveness. Collectively, our data indicate that a previous *C. pneumoniae* infection can affect IL-10 secretion by PBMCs in asthmatics and raise further demand to analyze immune response differences between steroid-sensitive and -resistant patients. 

Related to the TNF-α response, in this study, we found only one significant difference in *C. pneumoniae* seropositive asthmatics. PBMCs from steroid-resistant patients spontaneously secreted significantly higher levels of TNF-α than did those of steroid-sensitive patients, however, under *C. pneumoniae* stimulation no significant differences were detected between healthy volunteers, steroid-sensitive, and steroid resistant asthmatics. In contrast, pediatric patients with asthma have demonstrated a possibly altered ineffective Th1 immune response, resulting in lower TNF-α responses from *C. pneumoniae* infected PBMCs [29]. Differences among adult asthmatics have also been demonstrated under specific circumstances such as pregnancy [30]. These findings point out the possibility of impaired cytokine responses under determined factors that can interfere with asthma pathogenesis and *C. pneumoniae* infection [31].

Undoubtedly, former studies have revealed that TNF-α plays a central role in the pathogenesis of refractory asthma, particularly, in the remodeling process and steroid responsiveness. A higher TNF-α level was observed in BAL fluid and in the peripheral blood in asthmatics as compared with healthy controls [32]. The TNF-α axis contributes to activation of NF-κB, and therefore promotes proinflammatory cytokine expression. TNF-α induces the recruitment of eosinophil and neutrophil cells and plays a role in airway remodeling and decreased glucocorticoid response via cellular and immune responses [16,33]. *C. pneumoniae* is also involved in refractory asthma, as it alters the apoptosis process of infected cells and prolongs cell survival leading to airway structure changes. Cho et al. demonstrated that *C. pneumoniae*-infected PBMCs promoted cell proliferation in a Th2 microenvironment and T lymphocytes were resistant to the proapoptotic effect of glucocorticoids. These results were attributable to TNF-α axis activation and its modified function [18].

It is well-known that MMPs are involved in extracellular matrix changes and cytokine regulation. Moreover, MMP-9 levels correlate with lung function parameters, for example, FEV1 [34]. It is worth noting that pivotal differences in exhaled MMP-9 levels were detected in mild/moderate eosinophilic, severe eosinophilic, and severe neutrophilic asthmatics, indicating an association between asthma severity and airway remodeling [24].

The effect of corticosteroids on MMP-9 production is controversial. Inhaled steroids did not reduce the exhaled MMP-9 rate [34], and this trend was also observed in BAL fluids of steroid responder and non-responder patients with asthma [35]. A previous study revealed no difference between *C. pneumoniae* infected and uninfected PBMCs regarding MMP-9 production. However, *C. pneumoniae* infection increased TIMP-1 secretion, leading to a decreased MMP-9/TIMP-1 ratio. Moreover, dexamethasone treatment resulted in a further reduction of this ratio [22]. In this study, we measured the serum MMP-9 levels to define differences related to the *C. pneumoniae-*specific IgG serostatus and steroid responsiveness. Steroid-resistant *C. pneumoniae* seropositive patients had a lower MMP-9 level than did seronegative patients. In addition, *C. pneumoniae* seronegative steroid-resistant patients had significantly higher MMP-9 levels than did steroid-sensitive individuals, indicating the probable intensified remodeling process. Former studies have revealed a strong association between MMP-9 expression and *C. pneumoniae* infection in atherosclerotic plaques; moreover, Paolillo et al. observed increased MMP-9 production in *C. pneumoniae*-infected human endothelial cells [36,37]. In addition, it is well-known that MMP-9 plays a detrimental role in the pathogenesis of allergic airway diseases [38,39]. Nevertheless, the punctual effect of *C. pneumoniae* on MMP-9 production in bronchial epithelial cells and alveolar macrophages remains unclear and further studies are needed to define the role of *C. pneumoniae *in MMP-9 secretion and lung fibrosis. 

Taken together, our findings revealed unknown features of asthmatic patients and strengthened the line of evidence that former infection could affect asthma mechanisms. To the best of our knowledge, this is the first study that compared asthmatic patients on the basis of steroid responsiveness and *C. pneumoniae* seropositivity. In summary, we emphasize the following milestone results: (i) In steroid-sensitive *C. pneumoniae* positive patients a significant IL-10 production was observed as compared with control individuals; (ii) similar differences were found in spontaneous IL-10 secretion of PBMCs from seropositive steroid-resistant asthmatics; (iii) like resistant asthmatics, we found a significantly higher IL-10 production without treatment and under *C. pneumoniae* stimuli in seropositive patients as compared with seronegative individuals; (iv) additionally, this study raised that seropositive steroid-resistant asthmatics expressed a significantly higher level of spontaneous TNF-α than steroid-sensitive asthmatics; (v) significantly higher MMP-9 levels were found in steroid-resistant seronegative patients than seropositive patients; and (vi) the same trend was also seen as compared with seronegative steroid-sensitive participants. 

## 4. Materials and Methods 

### 4.1. Study Population and Participants

Eighty adult patients with asthma were recruited from the outpatient departments and inpatient wards at the Department of Pulmonology (University of Szeged, Hospital of Chest Diseases, Deszk). The inclusion criteria included clinically stable asthma, persistent asthma symptoms, inhaled steroid use, absence of current exacerbation, and complete follow-up periods. The exclusion criteria included a history of HIV infection, current viral or bacterial infections, chronic immunosuppression or autoimmune disease, cancer, systemic intravenous corticosteroid use (in the past 30 days), and antibiotic treatment (in the past 30 days). As a control group, 40 non-asthmatic, healthy blood donors without obstructive lung diseases, nasal polyposis, allergic rhinoconjunctivitis, cancer, chronic heart disease, autoimmune diseases, and immunosuppression were selected. Patients’ demographic and clinical characteristics were recorded. To investigate cytokine production, 5 mL native and 5 mL unfractionated heparin anticoagulated blood samples were collected from each patient. Before collecting blood samples from the patients with asthma, post-bronchodilator tests were performed. After administering 400 μg inhaled salbutamol, dynamic lung volumes (the FEV1, forced vital capacity [FVC], FEV1/FVC, and forced expiratory flow at 25% to 75% of the pulmonary volume [FEF25/75]) were measured. Spirometry was carried out using a Carefusion MasterScreen Body Plethysmograph (Sentrysuite software 2.13).

Asthmatic and control patients were not involved in the development, implementation, and interpretation of the study. Our study was undertaken in accordance with the Regional Human Biomedical Research Ethics Committee, University of Szeged (WHO-3220, 77/2013, 27/05/2013). Patients received written and verbal information about the purpose of blood sampling. All patients volunteered and their written informed consents were obtained. 

### 4.2. C. pneumoniae-Specific Enzyme-Linked Immunosorbent Assay

*C. pneumoniae*-specific antibodies from the patients and the controls were detected using the “NovaLisa TM Chlamydia pneumoniae” enzyme-linked immunosorbent assay [ELISA] kit (Nova Tec Immundiagnostica GmbH, Germany). Fifty-fold diluted sera were tested in duplicate in accordance with the manufacturer’s instructions for the presence of *C. pneumoniae*-specific immunoglobulin (Ig) G.

### 4.3. Preparation of the C. pneumoniae Antigen

*C. pneumoniae* CWL29 (ATCC, US) elementary bodies (EBs) were purified from infected Hep2 cells (ECACC, London, UK) by density gradient centrifugation and inactivated with formaldehyde treatment, as described by Penttila et al. [40]. The protein content of the antigen was measured by spectrophotometry, and the antigen was stored at −80 °C until use. 

### 4.4. Separation and Stimulation of PBMCs

PBMCs from 10 mL heparinized blood were separated using Ficoll gradient (Sigma), 5 × 10^5^ cells in three parallel wells were incubated in the presence of 2 μg/mL *C. pneumoniae* antigen or 10 μg/mL polyclonal mitogen (phytohemagglutinin, PHA) or left untreated in 200 μL RPMI medium containing 10% fetal bovine serum supplemented with glutamine, non-essential amino acids, gentamycin, and fluconazole. Supernatants of the stimulated wells were harvested 48 h after treatment, aliquoted, and stored at −80 °C until performing the cytokine ELISA.

### 4.5. Cytokine ELISA

The supernatants of the stimulated or untreated PBMCs were centrifuged (5 min, 1200 rpm) and assayed for the concentrations of IL-10 and TNF-α using Human Mini ELISA Development cytokine kits (PeproTech), while the quantity of MMP-9 in the sera was determined using the human MMP-9 ELISA kit (Sigma). The sensitivities of the IL-10, TNF-α, and MMP-9 measurements were in the range of 23 to 3000, 16 to 2000, and 8.23 to 6000 pg/mL, respectively. The clarified supernatants and sera were tested in duplicate in accordance with the manufacturer’s instructions.

### 4.6. Statistical Analysis 

Statistical analysis of the data was carried out using SigmaPlot for Windows Version 11.0 software, using the Wilcoxon–Mann–Whitney two-sample test. Differences were considered statistically significant at* p *< 0.05.

## 5. Conclusions

In summary, this study provides novel data about the different cytokine secretion of PBMCs from steroid-resistant and -sensitive asthmatic patients with or without stimulation. Our findings suggest that steroid resistance is associated with altered cytokine production based on *Chlamydia pneumoniae* serostatus. Our investigations support the heterogeneous features of asthma disease and contribute to define better steroid-resistant asthmatic characteristics at the cytokine level. Currently, there is increasing demand to use immunotherapy in asthma management based on the inhibition of Th2 cytokines or IgE [41,42]. Consequently, there is an emerging need to determine the immunological phenotypes of asthma, and therefore understand the pitfalls of conventional therapies. As our research was not without limitations, further studies are required to define the precise mechanisms underlying infection-mediated asthma and the long-term effect of persistent *C. pneumoniae* infection. 

## Figures and Tables

**Table 1:** Main clinical characteristics and demographic data of the steroid sensitive and steroid-resistant patients.

	**Steroid-sensitive n = 40**	**Steroid-resistant n = 40**	***p* Value**
**Mean age (median)**	59 (63)	63 (67)	0.13
**Gender**	male: 14 (35%), female: 26 (65%)	male: 13 (32%), female: 27 (68%)	-
**Smoking, mean py**	n = 15 (24, 5)	n = 19 (31)	0.14
**Smoking status**	Never: 25 Previously: 7 Habitual: 8	Never: 21 Previously: 10 Habitual: 9	
**Atopic subjects (allergic rhinitis, sinusitis, nasal polyposis)**	n = 22 (55%)	n = 25 (63%)	-
**CHD**	n = 2 (5%)	n = 17 (42%)	-
**Blood eosinophilia**	n = 11 (27%)	n = 17 (42%)	0.319
**FEV1 (L, %)**	2.1 ± 0.8 (72 ± 0.2%)	1.4 ± 0.6 (56 ± 0.2%)	0.01
**FEV1 reversibility rate** **(mean, %)**	15.2 ± 2.6	12.6 ± 3.3	0.41
**FVC (L, %)**	3.3 ± 1.1 (92.8 ± 24%)	2.5 ± 0.9 (79.9 ± 21%)	0.03
**FEV1/FVC (%)**	65.4 ± 11.3%	60.1 ± 14.8%	0.09
**FEF25/75 (L/s)**	1.4 ± 0.8	0.8 ± 0.4	0.004
**BMI (kg/m^2^)**	29.4 ± 8.1	27.6 ± 5.1	0.3
**Asthma medications**	ICS: 8 patients ICS/LABA: 32 patients SABA: 28 patients LTI: 20 patients	ICS/LABA: 40 patients LAMA: 8 patients SABA: 31 patients LTI: 27 patients	
